# The Molecular Chaperone DNAJB6, but Not DNAJB1, Suppresses the Seeded Aggregation of Alpha-Synuclein in Cells

**DOI:** 10.3390/ijms20184495

**Published:** 2019-09-11

**Authors:** Natasja Deshayes, Sertan Arkan, Christian Hansen

**Affiliations:** Molecular Neurobiology, Department of Experimental Medical Science, BMC B11, Lund University, 221 84 Lund, Sweden; natasja.deshayes@gmail.com (N.D.); sertan.arkan@med.lu.se (S.A.)

**Keywords:** DNAJ protein, co-chaperone alpha-synuclein, Parkinson’s disease

## Abstract

Alpha-synuclein (α-Syn) can misfold and aggregate, causing the degeneration of dopaminergic neurons, as seen in Parkinson’s disease (PD). We recently demonstrated that DNAJB6, a co-chaperone found in Lewy bodies (LB), suppresses the aggregation of α-Syn in cells and in vitro. In this study, we compared the capacities of DNAJB1 and DNAJB6 to suppress the seeded α-Syn aggregation in HEK293 cells expressing α-Syn tagged with cyan fluorescent protein (CFP) or yellow fluorescent protein (YFP). The aggregation of α-Syn was seeded by the transfection of the cells with recombinant α-Syn pre-formed fibrils (PFFs), following the Clustered Regularly Interspaced Short Palindromic Repeats (CRISPR)-Cas9-mediated knockout (KO) of these two genes, respectively. We quantified the α-Syn aggregation by fluorescence microscopy and fluorescence resonance energy transfer (FRET) analysis. We detected significantly more aggregates in the *DNAJB6* KO cells compared with the parental cells, whereas the *DNAJB1* KO had no effect on the α-Syn aggregation. This is the first evidence that DNAJB6 can suppress α-Syn aggregation, induced by exogenous α-Syn seeds, in cells. Next, we explored whether this mechanism could be dependent on protein degradation pathways. We observed that the increase in the α-Syn PFF-induced aggregation in the *DNAJB6* KO cells compared with the parental cells was strongly diminished upon the incubation of the cells with the proteasomal inhibitor MG132. These results consolidate that DNAJB6 is a suppressor of α-Syn aggregation, and suggest that DNAJB6 may target misfolded and/or aggregated α-Syn for proteasomal degradation.

## 1. Introduction

Parkinson’s disease (PD) is the second most common neurodegenerative disorder [1,2], affecting millions of people worldwide [3]. The classical motor symptoms of PD, including tremors, rigidity, and bradykinesia, are mainly as a result of the degeneration of the dopaminergic neurons in the *substantia nigra pars compacta* [1,4,5,6]. A hallmark of PD is the presence of Lewy bodies in neurons [3]. These are cytoplasmic protein-rich inclusions [2], in which α-Synuclein (α-Syn) is the most abundant protein [4].

α-Syn is widely expressed in the neurons of the brain, and is mainly localized at the presynaptic terminals, where it is suggested to play a role in the synaptic vesicle dynamics and dopaminergic neurotransmission [3,7]. Under physiological circumstances, α-Syn is an unfolded monomer of 140 amino acids [3,8]. When it is misfolded, monomeric α-Syn can oligomerize into toxic oligomers and protofibrils that are able to form larger fibrils, which, in turn, may accumulate into Lewy bodies and Lewy neurites [3,9].

The multiplication of and several missense mutations in the α-Syn-encoding gene have been linked to familial PD [3,4,7]. However, the exact cause of sporadic PD, which accounts for more than 85% of the cases [3,10], remains largely elusive [11].

The misfolding and aggregation of α-Syn may be the result of an age-dependent impairment of the cellular mechanisms that work to maintain proteostasis [5], in which molecular chaperones play a crucial role [6]. Heat shock proteins of 70 kDa (Hsp70) are protective against protein aggregation [12], and a series of in vitro and in vivo studies have indeed demonstrated that Hsp70 can ameliorate α-Syn aggregation [13] and α-Syn-induced neurotoxicity [2,14]. Hsp70 members promote the proper folding of non-native proteins in an ATP-dependent cycle, which is regulated by DNAJ/Hsp40 co-chaperones (hereafter named DNAJ proteins) [15] and nucleotide exchange factors (NEFs) [10]. The DNAJ proteins are responsible for the recognition of and binding to misfolded polypeptides, after which they confer to Hsp70 chaperones, which in turn promote either the folding of the polypeptide into its native structure or degradation [15,16].

There are more than 40 DNAJ proteins encoded in the human genome [15,17], of which the majority are expressed in the brain [18]. The DNAJ protein DNAJB6 has previously been found to suppress the aggregation of multiple amyloid proteins. Several studies have confirmed that DNAJB6, when overexpressed in cells or in a mouse model, is able to suppress the aggregation of expanded polyglutamine (polyQ) or exon 1, with the expanded polyQ of Huntingtin (Htt) [19,20,21,22]. Other studies have found that it inhibits the aggregation of amyloid beta in cells and in vitro as well [23,24,25]. DNAJB6 is expressed in several cell types in the brain, including in the neurons, and is also found to be present in the Lewy bodies of PD patients [26]. Our research group recently demonstrated that the knockout (KO) of DNAB6 in cells increases the α-Syn aggregation, and that DNAJB6 delays α-Syn aggregation in vitro [27], which strongly suggests an important role of DNAJB6 in preventing α-Syn aggregates from accumulating in vivo.

Another DNAJ protein that is abundantly expressed in the brain, DNAJB1 [28], is known to be involved in the degradation of polyglutamine proteins [28,29], and the reduction of Htt aggregation in Huntington’s disease [30]. Moreover, Gao et al. demonstrated that DNAJB1 is part of a complex that is able to solubilize α-Syn fibrils to monomers, thereby linking DNAJB1 to α-Syn aggregation [13]. However, this was observed in a cell-free system, rendering this role of DNAJB1 in cells elusive. Therefore, the aim of this study was to compare the capacities of DNAJB1 and DNAJB6 to suppress the aggregation of α-Syn, using an unbiased fluorescence resonance energy transfer (FRET)-based technique to quantify this in a human embryonic kidney 293 (HEK293) cell line. Here, we generated KOs of these two genes in the HEK293 cells by CRISPR-Cas9, and demonstrate that DNAJB6, but not DNAJB1, suppresses the aggregation of α-Syn in cells.

## 2. Results

**Figure 1:**
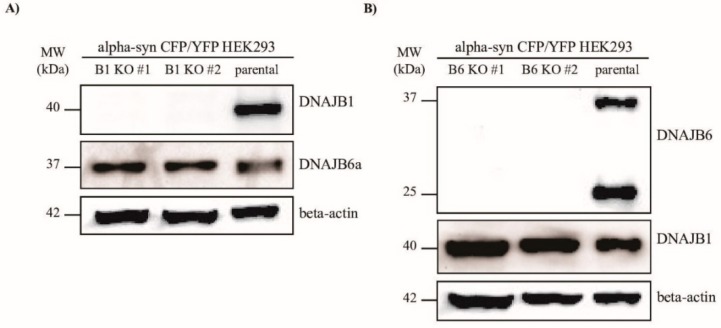
Western blot analysis of the CRISPR-Cas9-mediated knockout (KO) of *DNAJB1* and *DNAJB6* in the alpha-synuclein (α-Syn) CFP/YFP HEK293 cells. Western blots displaying the expression of (**A**) DNAJB1 or (**B**) DNAJB6 in the cell lines knocked out for these two genes, respectively, or parental control α-Syn CFP/YFP HEK293 cells.

To examine whether DNAJB1 and DNAJB6 could modulate the aggregation of α-Syn, we knocked out the genes encoding these two proteins, respectively, in the HEK293 cells, that constitutively express α-Syn fused to either CFP or YFP. In this cell line, which was a kind gift from Marc Diamond’s lab [31], the α-Syn aggregates could be measured quantitatively by FRET, or detected by fluorescence microscopy. Using the CRISPR–Cas9 technology, two KO clones for each of the *DNAJB1* and *DNAJB6* genes were generated (Figure 1A,B).

**Figure 2:**
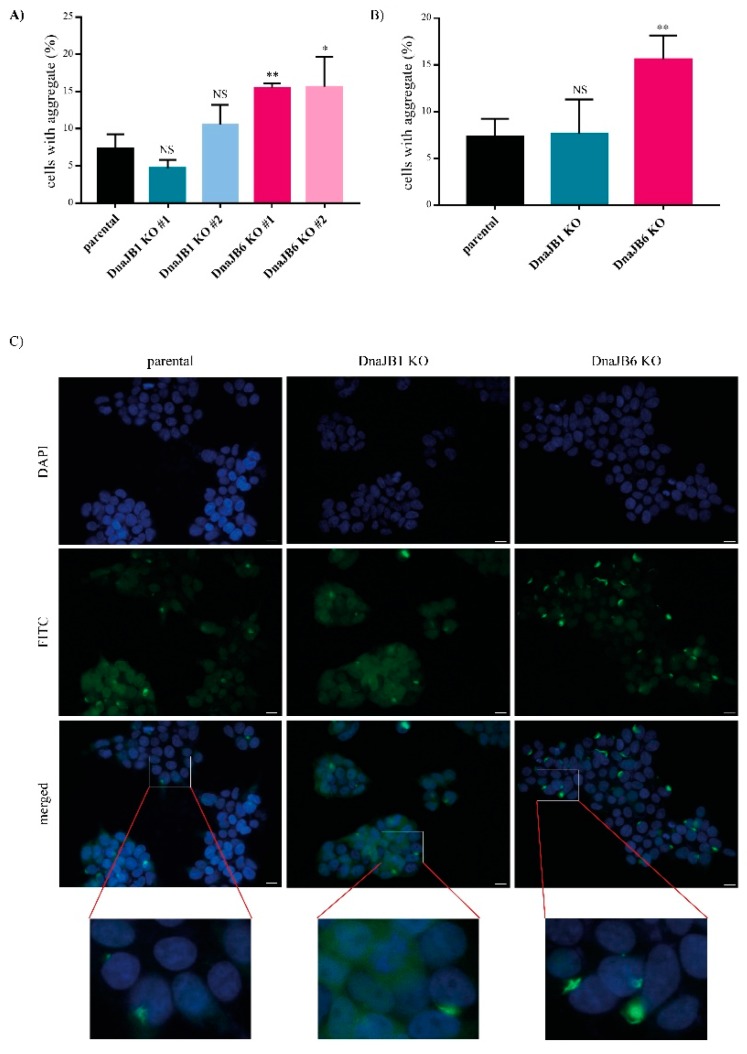
α-Syn pre-formed fibrils (PFFs)-induced aggregation in *DNAJB1* KO, *DNAJB6* KO, or parental control α-Syn CFP/YFP HEK293 cells, analyzed by immunofluorescence microscopy. (**A**) Quantifications of the percentage of cells positive for fluorescent aggregates in parental cells (7.36 ± 1.07), and two different individual clones of *DNAJB1* (4.68 ± 0.629 and 10.5 ± 1.53) or *DNAJB6* KO (15.5 ± 0.31 and 15.6 ± 2.3), respectively. (**B**) These numbers were averaged for the two independent clones of each KO (7.61 ± 1.5 and 15.6 ± 1.04), respectively. The data were analyzed by the Student’s *t*-test, and are represented as mean ± standard deviation (SD), namely: ns *p* > 0.05 * *p* < 0.05; ** *p* < 0.01 (*n* = 3). (**C**) Representative pictures of DAPI stained of parental, *DNAJB1* KO, and *DNAJB6* KO α-Syn CFP/YFP HEK293 cells. The cells were transfected with 10 nM α-Syn PFFs for 48 h, prior to these analyses. Scalebar: 20 µm.

In order to induce α-Syn aggregation in these KO clones, 10 nM of pre-formed fibrils (PFFs) of α-Syn were added to the cells for 48 h. The aggregation of α-Syn was first assessed by IF microscopy, and was determined by counting the percentage of cells showing a combination of CFP- and YFP- positive aggregates. The *DNAJB6* KO cells showed a two-fold increase in the α-Syn aggregation compared with the parental cells for both the individual *DNAJB6* KO clones, whereas none of the *DNAJB1* KO clones displayed any significant change in the α-Syn aggregation compared with the parental cells (Figure 2A). On average, for the independent clones, *DNAJB6* KO resulted in a strong, significant increase in α-Syn aggregation (Figure 2B,C). Staining for the expression of endogenous DNAJB1 and DNAJB6 revealed that both are, to a large extend, found in the cytoplasm, and therefore colocalize in the same compartment as α-Syn (Supplementary Figures S1 and S2).

**Figure 3:**
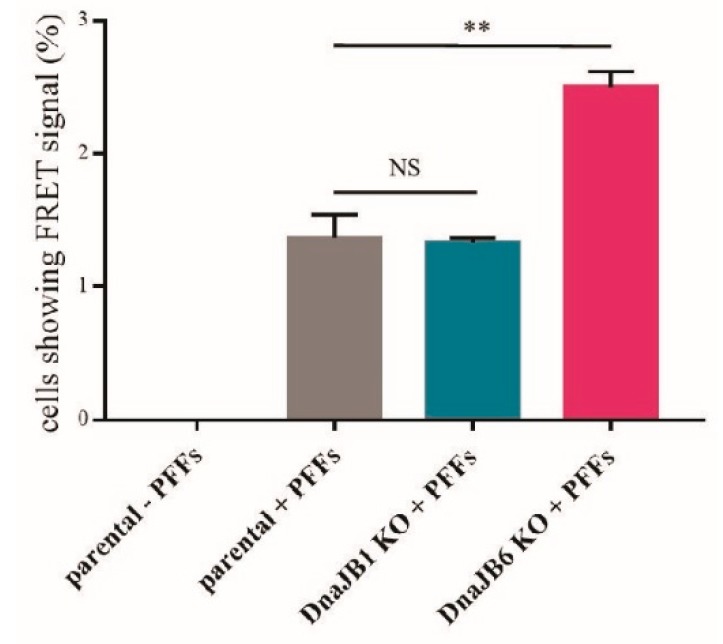
The α-Syn PFF-induced aggregation of α-Syn following the KO of *DNAJB1* or *DNAJB6* in the α-Syn CFP/YFP HEK293 cells analyzed by fluorescence resonance energy transfer (FRET). The quantification of the percentage of alive and single α-Syn CFP/YFP HEK293 cells producing a FRET signal, as detected by BD LSRFortessa™. The cells had been transfected with 10 nM of α-Syn PFFs for 48 h, ahead of the experiment. The data were analyzed by the Student’s *t*-test, and are represented as mean ± SD: ns *p* > 0.05 * *p* < 0.05; ** *p* < 0.01 (*n* = 3; 0.00; 1.37 ± 0.176; 1.33 ± 0.0333; 2.5 ± 0.115).

To verify the results obtained by the fluorescence microscopy, a FRET analysis was performed. In this experiment, the percentage of single FRET-positive cells was measured. As previously demonstrated by Marc Diamond and colleagues, in α-Syn-CFP/YFP HEK293 cells, a FRET signal is only detected upon the induction of α-Syn aggregation by α-Syn PFFs [31]. As expected, there was no FRET signal observed in the cells that had not been transfected with α-Syn PFFs (Figure 3). In accordance with the results from the fluorescence microscopy experiment, there was no difference in the percentage of FRET-positive cells between the *DNAJB1* KO and parental cells that had been transfected with α-Syn PFFs. However, the percentage of FRET-positive *DNAJB6* KO cells was strongly increased in comparison with the percentage of FRET-positive parental cells that had been transfected with α-Syn PFFs. These results were in line with the previously published data from our lab, demonstrating that DNAJB6 is a suppressor of α-Syn aggregation [27]. In addition, this showed that DNAJB6 can suppress the α-Syn aggregation, which is seeded by exogenous fibrils (Figure 3 and Supplementary Figure S3).

A large body of work has shown that the DNAJ proteins can assist the Hsp70 proteins in either refolding the unfolded and misfolded proteins, or targeting these proteins for degradation. There is evidence in the literature that the misfolded α-Syn can be targeted for degradation, via both the ubiquitin–protease and autophagy–lysosome pathways [32,33,34]. To explore whether DNAJB6 might be particularly relevant for targeting α-Syn for either lysosomal or proteasomal degradation, we induced α-Syn aggregation in the parental and *DNAJB6* KO α-Syn FRET cells using α-Syn PFFs, while at the same time inhibiting either the lysosomal or proteasomal activity by treating the cells with bafilomycin A or MG132, respectively. Next, the effect of bafilomycin A and MG132 on α-Syn aggregation in the parental and *DNAJB6* KO cells was analyzed by fluorescence microscopy, as well as by FRET analysis.

**Figure 4:**
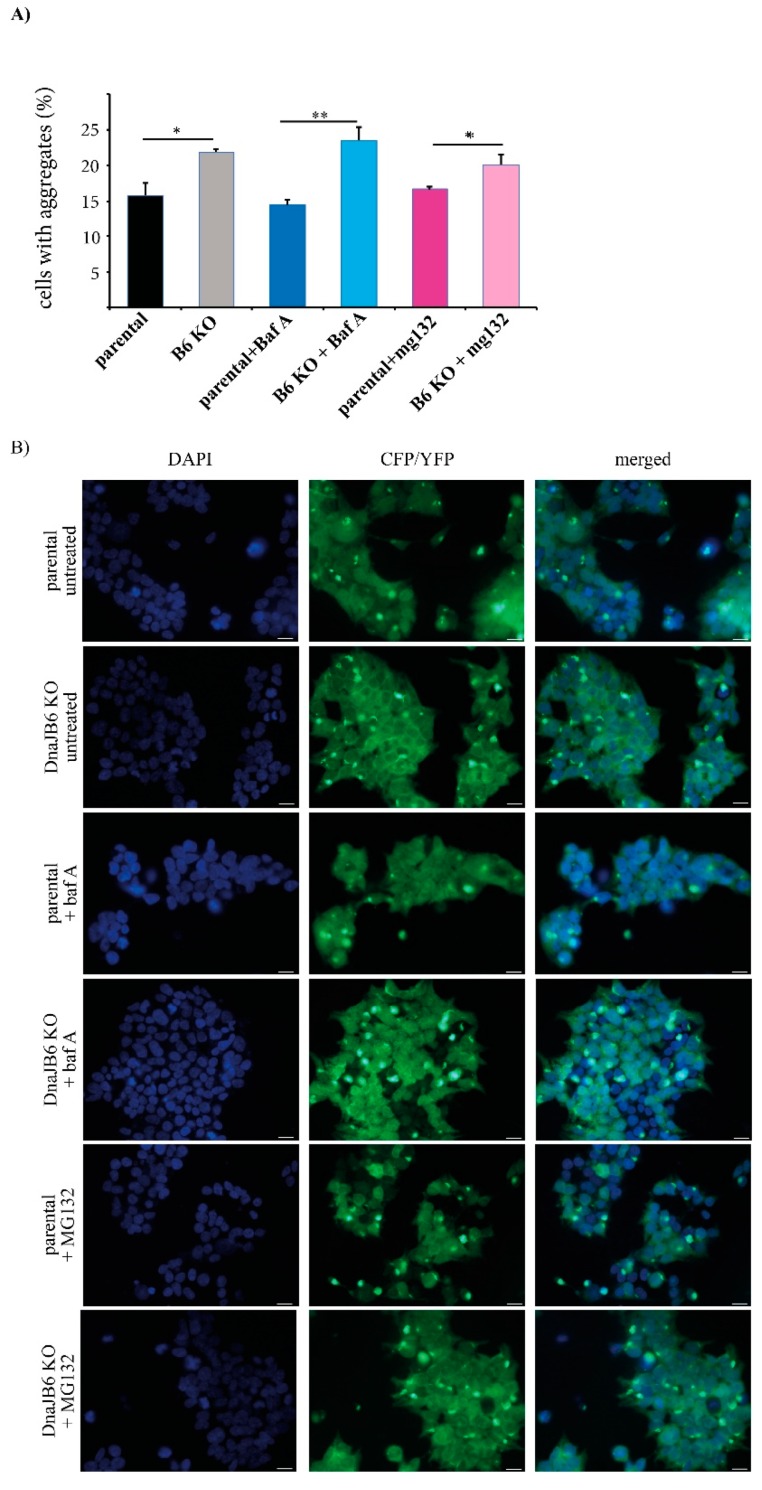
Effect of the lysosomal or proteasomal inhibitor treatment on the α-Syn PFF-induced aggregation of α-Syn in α-Syn CFP/YFP HEK293 cells analyzed by immunofluorescence microscopy. (**A**) Quantification of the percentage of DAPI-positive parental and *DNAJB6* KO cells displaying CFP/YFP positive aggregates as counted using flourescence microscopy (15.8 ± 0.962; 21.9 ± 0.233; 14.5 ± 0.472; 23.5 ± 1.11; 16.6 ± 0.218; 20.1 ± 0.837). The cells were transfected with 10 nM α-Syn PFFs for 48 h, and treated (or not) with 100 nM of bafilomycin A or 25 µM of MG132 for 2 h. (**B**) Representative pictures of the fixed parental and *DNAJB6* KO α-Syn FRET HEK293 cells and DAPI stained after three days of culture following transfection with 10 nM of α-Syn PFFs for 48 h. The cells were either not treated (untreated), or treated with 100 nM bafilomycin A for 2 h or 25 µM MG132 for 2 h. Magnification 40×. Data were analyzed by the Student’s *t*-test and are represented as mean ± SD: * *p* <0.05; ** *p* <0.01 (*n* = 3). Scalebar: 20 µm.

The fluorescence microscopy results showed that there were significantly more α-Syn aggregates in the *DNAJB6* KO cells compared with the parental cells that were not treated (average increase 38%) or incubated with the lysosomal inhibitor (average increase 65%), bafilomycin A, whereas there was a much weaker difference in the α-Syn aggregation between the *DNAJB6* KO and parental cells that had been incubated with the proteasomal inhibitor MG132 (average increase 20%; Figure 4A,B).

**Figure 5:**
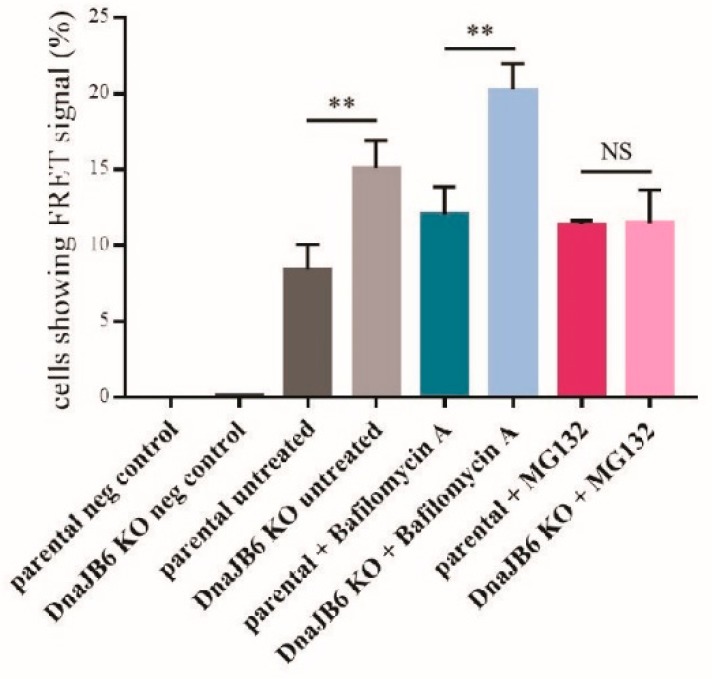
The α-Syn PFF-induced aggregation of α-Syn following lysosomal or proteasomal inhibitor treatment in α-Syn CFP/YFP HEK293 cells analyzed by FRET. Quantification of the percentage of alive and single α-Syn FRET HEK293 cells producing a FRET signal, as detected by BD LSRFortessa™. The cells had been transfected with 10 nM α-Syn PFFs for 48 h, and were treated (or not) with 100 nM bafilomycin A for 2 h or 25 µM MG132 for 2 h. The data were analyzed by the Student’s *t*-test and are represented as mean ± SD: ns *p* > 0.05 * *p* < 0.05; ** *p* < 0.01 (*n* = 3); 0.0; 0.1; 8.4 ± 0.95; 15.1 ± 1.06; 12 ± 1.05; 20.3 ± 0.984; 11.4 ± 0.145; 11.5 ± 1.24).

Next, we repeated these experiments, while using an unbiased FRET signal as a readout instead. Using this setup, we observed that upon incubation with the proteasomal inhibitor MG132, the effect of *DNAJB6* KO on α-Syn aggregation compared with the parental cells, was completely blocked. When we analyzed the effect of the *DNAJB6* KO cells in the conditions of the non-treated cells or the cells treated with the lysosomal inhibitor, we observed that the *DNAJB6* KO cells still had much more aggregates than the parental cells (79% and 68% average increase, respectively; Figure 5). These observations suggest that DNAJB6 may normally facilitate in the degradation of misfolded α-Syn via the proteasomal pathway.

## 3. Discussion

PD pathology is strongly linked to the aggregation of misfolded α-Syn. The aggregation of misfolded proteins is normally counteracted by cellular protein quality control mechanisms. An important role in this system is played by Hsps, including DNAJ proteins. In previous studies, we showed that DNAJB6 suppress the aggregation of α-Syn in cells and in vitro [27]. In this study, we analyzed the propensities of two different DNAJ proteins, DNAJB1 and DNAJB6, to suppress α-Syn aggregation, by counting the aggregates using IF microscopy, as well as by FRET analysis.

Instead of overexpressing the genes encoding DNAJB1 and DNAJB6, which can cause unspecific effects, we chose to generate KOs of the genes in the HEK293 cells by creating indel mutations, using the CRISPR-Cas9 technology (Figure 1). In order to study the effect of *DNAJB1* and *DNAJB6* KO on α-Syn aggregation, aggregation had to be induced. The aggregation of endogenous α-Syn was triggered by the incubation of the cells with 10 nM of α-Syn PFFs for 48 h. This way of induction of α-Syn aggregation is well-established [35], and is in line with the Braak hypothesis, which assumes that misfolded α-Syn spreads in a prion-like manner, and is able to induce the aggregation of endogenous α-Syn in recipient neurons [36].

The effect of *DNAJB1* and *DNAJB6* KO on α-Syn aggregation was first determined by counting the percentage of cells that contain aggregates, using fluorescence microscopy. Whereas none of the *DNAJB1* KO clones affected α-Syn aggregation, when compared with the parental cells, both the *DnaJB6* KO clones showed an increased number of α-Syn aggregates (Figure 2). The effect of DnaJB6 KO is in line with previous data from our lab, which demonstrated that DNAJB6 suppresses α-Syn aggregation in vitro as well as in cells [27]. However, DNAJB1 has found to be able to solubilize α-Syn fibrils to α-Syn monomers [13]. The contrast between this and our present study might be explained by the fact that the study by Gao and colleagues was performed in vitro, whereas we used a cellular experimental set-up.

In addition to fluorescence microscopy, we performed a FRET analysis to detect the aggregation of α-Syn. In order to use the FRET signal as a measure of α-Syn aggregation, we used the α-Syn-CFP/YFP HEK293 cell line, which was developed in the Diamond lab [31]. Again, we observed that the *DNAJB1* KO had no effect on the α-Syn aggregation, while the *DNAJB6* KO increased the α-Syn aggregation, in comparison to the parental cells. These results not only verified the fluorescence microscopy results, but also indicate that this FRET-based system can be used to quantitatively study α-Syn aggregation in an unbiased way (Figure 3). α-Syn is mainly localized to the cytoplasm, as are DNAJB1 and DNAJB6 (Supplementary Figures S1 and S2). Therefore, the effect of DNAJB6 as a suppressor of α-Syn aggregation cannot be explained by cellular localization alone.

An important function of the cellular chaperone machinery is to detect the misfolded and/or aggregated proteins, and, if they cannot be refolded, to target these for degradation. To study if the α-Syn aggregation suppressor, DNAJB6, may be important in such a mechanism, we incubated the parental and *DNAJB6* KO cells with or without a lysosomal (bafilomycin A) or proteasomal (MG132) inhibitor for 2 h. We observed a consistent significant increase in α-Syn aggregation in *DNAJB6* KO cells, compared to parental cells, but this increase was either strongly diminished (fluorescence microscopy read out) or completely abolished (FRET analysis) when the cells were incubated with the proteasomal inhibitor (Figure 4 and Figure 5). Therefore, these results indicate that DNAJB6 may be important for detecting misfolded α-Syn, and targeting it for degradation via the proteasome. As DNAJB6, α-Syn, and proteasome are all found, to a large extend, in the cytoplasm (Supplementary Figures S1 and S4), then this goes well in line with this hypothesis. The results from the fluorescence microscopy counting and FRET analysis were very similar with regards to the ratios of aggregates between the parental and KO cells, and under the conditions of incubating the cells with inhibitors. However, the small differences that were found could be explained by the fact that in the fluorescence microscopy, we used a manual quantification of cells that had one aggregate or more aggregates, and in the FRET, the size of the aggregate that give rise to a FRET signal might be different, as this could, in principle, be caused by smaller oligomers.

The increase in the total amount of aggregates in the cells incubated with the lysosomal or proteosomal inhibitor compared to the control cells was non-existent or very subtle in the different experimental settings. However, we believe that the short timespan used for incubation with these inhibitors (few hours) may not be sufficient to increase the amount of aggregates. Also, it has been suggested that the α-Syn aggregates may both be degraded via the proteasomal degradation and autophagy, and therefore, when using one inhibitor, an increased degradation via another pathway might take place.

The overexpression of DNAJB6 does not lower the total levels of the α-Syn in the HEK293 cells overexpressing α-Syn [27] but it is possible that only a small portion of α-Syn is misfolded or aggregated, and that the targeting of the degradation of these aggregates is facilitated by DNAJB6.

So far, DNAJB6 is the only DNAJ protein known to suppress α-Syn aggregation. However, several other DNAJ proteins, including DNAJB2, DNAJC6, and DNAJC13, have been genetically linked to PD [37,38,39,40]. It will be interesting to explore whether these DNAJ proteins are also somehow linked to α-Syn aggregation, or if their genetic link to PD is completely independent of α-Syn aggregation. Future studies should uncover whether the α-Syn aggregation suppressor property is unique to DNAJB6, or whether it is shared by other DNAJ proteins, which are perhaps required for this in different cell types or cellular compartments, as α-Syn is present in most cellular compartments [3].

As this and other studies have shown that DNAJB6 can suppress both α-Syn and polyQ aggregation, it is possible that it suppresses the aggregation of these proteins in a mechanistically similar manner. In the DNAJ overexpression studies, when evaluating several DNAJ proteins for the potential to suppress polyQ aggregation in HEK293 cells, DNAJB8 was found to be an equally potent suppressor of polyQ aggregation as DNAJB6 [20,21]. However, as DNAJB8 is almost exclusively expressed in the testis [41], it is unlikely to play a protective role against the development of neurodegenerative diseases.

Very little work has so far addressed the potential role of DNAJ proteins as suppressors of amyloid aggregation in neurodegenerative diseases. However, one study has established that DNAJB6 can suppress the aggregation of the amyloid protein in an animal model of Huntington’s disease [19]. Future studies should address whether DNAJB6 is an equally potent suppressor of PD progression in α-Syn overexpression-based animal models, and whether this DNAJ protein could be a target in the design of novel PD therapies.

## 4. Material and Methods

### 4.1. CRISPR/Cas9 Guide Design

The CRISPR/Cas9 guide sequences targeting DnaJB1 or DnaJB6 were designed using the following sanger program: http://www.sanger.ac.uk./htgt/wge/find_crisprs. The sequences used for the guides targeting the DNAJB1 gene were as follows: 5′caccgtctcctcgtccgacgcgccg3′, 5′aaaccggcgcgtcggacgaggaga3′, 5′caccgcgggtcgctgagcacgtcgt3′, and 5′aaacacgacgtgctcagcgacccgc3′. The sequences used for the guides targeting the DNAJB6 gene were as follows: 5′caccgttacgcctttttaatatcct 3′, 5′aaacaggatattaaaaaggcgtaac3′, 5′caccgggaggcatatgaagtgctgt3′, and 5′aaacacagcacttcatatgcctccc′3. The guides were cloned into two separate Cas9-mCherry constructs (derived from a Cas9-GFP construct, Addgene: cat#48138) and HEK293 cells constitutively expressing alpha-synuclein (α-Syn) fused to either CFP or YFP (α-Syn-CFP/YFP HEK293 cells), which were a kind gift from Marc Diamond’s lab [31], were subsequently transformed according to the protocols described in the literature [42]. After 36 h, the Cas9-mCherry-positive cells were single cell sorted into 96-wells plates by means of fluorescence activated cell sorting (FACS). The single cell clones were analyzed for the KO of DnaJB1 or DnaJB6 by Western blot analysis.

### 4.2. Cell Culture, Transfection, and Reagents

The α-Syn-CFP/YFP HEK293 cells were maintained in Dulbecco’s Modified Eagle’s Medium (DMEM) (Sigma-Aldrich, St. Louis, Missouri, USA) plus 10% fetal bovine serum (FBS; Thermo Fisher Scientific, Massachusetts, USA) and 1% penicillin/streptomycin (Thermo Fisher Scientific, Massachusetts, USA). Approximately 24 h after plating into 24-wells plates at 50,000 cells/well, the α-Syn-CFP/YFPHEK293 cells were transiently transfected with 10 nM of pre-formed fibrils (PFFs) of α-Syn using Lipofectamine 2000 (Invitrogene, Thermo Fisher Scientific, Massachusetts, USA) for 48 h to induce the aggregation of α-Syn. When the cells were incubated with inhibitors, this was performed with 100 nM Bafilomycin A (Sigma-Aldrich St. Louis, Missouri, USA) for 2 h, or 25 µM MG132 (Sigma-Aldrich) for 2 h at 37 °C until fixation or FRET assay preparation.

### 4.3. SDS-PAGE, Western Blotting and Antibodies

The cells were washed with PBS and subsequently lysed in a lysis buffer (0.5% Triton X-100, 50 mM Tris HCl, 175 mM NaCl and 5 mM Ethylenediaminetetraacetic acid (EDTA), pH 8, 1:100 protease inhibitor cocktail (Sigma-Aldrich; P8340, St. Louis, Missouri, USA)) on ice for at least 15 min. The debris was spun down at 15,000 rcf for 10 min at 4 °C, and the supernatant was collected, mixed with 1× Laemmli buffer plus 10% of 0.1M Dithiothreitol (DTT), and boiled for 5 min at 95 °C. The proteins (10 or 15 µg total) were separated on 8% or 10% SDS-PAGE polyacrylamide gels and subjected to Western blot transfer onto Polyvinylidene fluorid (PVDF) membranes using the Trans-Blot Turbo Transfer System (Bio-Rad, California, USA). The membranes were then blocked in 5% *w/v* skimmed milk powder dissolved in PBS containing 0.05% Tween (PBS-T) for 1 h at room temperature (RT), after which they were washed three times for 5 min with PBS-T at RT. Subsequently, the membranes were incubated with a primary antibody (1:2000 in PBS-T with 2% skimmed milk powder) on a shaker overnight at 4 °C. The next day, the membranes were again washed with PBS-T three times for 5 min at RT, followed by incubation with a secondary antibody (1:10,000 in PBS-T with 3% skimmed milk powder) for 60 min at RT. After applying a Western Blotting Luminol Reagent (Santa Cruz Biotechnologies Texas, USA) to the membrane, the detection of chemiluminescence was performed with the use of a ChemiDoc™ XRS + System (Bio-Rad, California, USA). The primary antibodies used were anti-DNAJB1 (Enzo Life Sciences) and anti-DNAJB6 (Abcam Cambridge, UK). The secondary antibody used was goat anti-rabbit-Horseradishperoxidase- (HRP)conjugated (Dako Glostrup, Denmark).

### 4.4. Fluorescence and Immunoflourescence Microscopy

Glass coverslips were coated with 0.1 mg/mL poly-D-lysine (Sigma-Aldrich, St. Louis, Missouri, USA) for 20 min, prior to the experiment. In each experiment, 50,000 cells were plated on a coverslip in a well of a 24-well plate, and incubated overnight. Subsequently, the cells were transfected with α-Syn PFFs for 48 h, and the fluorescence of CFP/YFP was performed to detect α-Syn aggregation. The α-Syn-CFP/YFP HEK293 cells were washed with PBS-T, fixed by incubation in 4% paraformaldehyde for 15 min, and washed with PBS-T thrice. The coverslips were mounted onto microscopic slides using a Vectashield^®^ mounting medium with DAPI (Vector Laboratories California, USA). Images were taken using the Plan Apo 40×/1.0 oil objective of a Nikon Eclipse 80i microscope and NIS-Elements imaging software. The nuclei were visualized in the DAPI channel, whereas the α-Syn aggregation was detected using the FITC channel. The brightness of the images was equally modified in ImageJ.

For the immunocytochemistry staining, the glass coverslips were coated with 0.1 mg/mL poly-D-lysine (Sigma Aldrich) for 20 min, before the beginning of the experiments. Next, 40,000 cells of either parental α-Syn FRET HEK293T, DNAJB1 KO, and DNAJB6 KO respectively, were each plated to wells of 24-well plates, containing coverslips, and allowed to incubate overnight. The cells were subsequently fixed with 4% paraformaldehyde for 20 min and washed with PBS thrice. Following, the cells were incubated with 0.5% Triton X-100 for 5 min (Sigma Aldrich St. Louis, Missouri, USA) and then washed with PBS thrice. After 1 h of blocking with 5% bovine serum albumin (Sigma Aldrich St. Louis, Missouri, USA), the cells were incubated with either anti-DNAJB1 (Enzo Life Sciences), anti-DNAJB6 (Abcam, Cambridge, UK), anti-proteasome 20S alpha and or beta-actin (Abcam, Cambridge, UK) primary antibodies for 1 h. After washing with PBS once, the cells were incubated with a donkey anti rabbit secondary antibody with Cy3 conjugated (Jackson ImmunoResearch, Philadelphia, USA) for 1 h in the dark. The coverslips were mounted onto microscopic slides using a Vectashield^®^ mounting medium with DAPI (Vector Laboratories California, USA). The images were taken using the Plan Apo 40×/1.0 oil objective of a Nikon Eclipse 80i microscope and NIS-Elements imaging software. The nuclei were visualized with DAPI staining, and α-Syn-CFP/YFP was detected by the CFP/YFP fluorescence, while the DNAJB1, DNAJB6, and proteasome 20S alpha and beta labeling of cells were detected by staining with cy3-labeled goat anti-rabbit antibody.

### 4.5. Aggregation Analysis

To calculate the percentage of cells displaying one or more aggregates, the total number of DAPI-positive cells in five different pictures (250 cells or more) were counted and analyzed for the presence of fluorescent aggregates (puncta) in ImageJ. For each condition, three coverslips were analyzed. To calculate the percentage of cells displaying an aggregate, the total number of DAPI-positive cells in five or six different pictures per coverslip (with a minimum of 287 and an average of 450 cells per picture) were counted and analyzed for the presence of YFP/CFP double-positive aggregates in ImageJ, using the cell counter plug-in. The total number of aggregates observed in the pictures belonging to one experiment was then divided by the total number of cells counted in the pictures from that experiment, and subsequently multiplied by 100. For each condition, three independent experiments were performed and analyzed (*n* = 3).

### 4.6. FRET Assay

Four days before the performance of the FRET biosensor assay, 50,000 to 70,000 α-Syn-CFP/YFPHEK293 cells were replated into 24-wells plates. Transfection with 10 nM of α-Syn PFFs took place 48 h in advance. On the day of the FRET assay, the cells were trypsinized and centrifuged for 2 min at 0.4 rcf. After the removal of the supernatant medium, the cells were resuspended in 100 to 200 ul DMEM and FBS and Pen/Strep. FRET was performed using BD LSRFortessa™ (BD Biosciences, New Jersey, USA). The CFP and FRET were measured by excitation using the 405 nm laser, and the fluorescence was captured with a 442/46 nm and 525/50 nm filter, respectively. For the measurement of the YFP, the cells were excited with the 488 nm laser and fluorescence was captured with the 530/30 nm filter. For each sample, up to 10,000 cells were analyzed for the FRET positivity, and each condition was examined in triplicate.

### 4.7. Preparation of α-Syn PFFs

The human α-Syn was expressed and purified as a monomeric fraction from an *E.coli* BL21 (DE3) gold strain, as previously described [27]. Monomeric α-Syn (70 μM, 300 μL) was incubated in 50 mM Tris pH 7.4, 150 mM KCl, and 5 mM MgCl2 (with 0.01% NaN3 to prevent bacterial growth) at 37 °C, under constant shaking at 200 rpm for four days. The fibrillar pellets were centrifuged (16,000× *g*, 30 min) and washed twice with a buffer (300 μL). The fibrils were resuspended at a concentration of 100 μM and sonicated (1 min, 10% max power, 30% cycles) using a probe sonicator (Bandelin, Sonopuls HD 2070, Bandelin Elec., Germany) in order to produce the first generation seeds. The second generation fibrils were prepared by incubating monomeric α-Syn (100 μM) in the presence of first generation seeds (10 μM) in 50 mM Tris pH 7.4, 150 mM KCl, 5 mM MgCl_2_, and 0.01% NaN_3_ (500 μL) at 37 °C under quiescent conditions for 13-14 h. The suspension was finally sonicated (20 s, 10% max power, 30% cycles).

### 4.8. Statistical Analysis

The statistical significance was determined/analyzed with Student’s *t*-tests using GraphPad Prism 7 software (San Diego, California, USA). Results with *p*-values of ≤ 0.05 were considered as statistically significant. The values are expressed as the mean ± standard deviation (SD).

If the data passed the normality test, according to the Shapiro-Wilk normality test, the statistical significance was determined with Student’s *t*-tests. If the data did not follow a normal distribution, a Mann-Whitney test was used to determine the statistical significance. All of the statistical tests were performed using GraphPad Prism 7 software (San Diego, California, USA). Results with *p*-values of ≤ 0.05 were considered as statistically significant. The values are expressed as the mean ± standard deviation.

## Supplementary Materials

Supplementary Materials can be found at https://www.mdpi.com/1422-0067/20/18/4495/s1.
