# Supplementary material for: The Molecular Chaperone DNAJB6, but Not DNAJB1, Suppresses the Seeded Aggregation of Alpha-Synuclein in Cells
Source: Int J Mol Sci. 2019 Sep 11;20(18):4495. doi: 10.3390/ijms20184495 (PMC6769935; doi:10.3390/ijms20184495)
Supplement: Supplementary file 1 [file ijms-20-04495-s001.pdf]

Supplementary figure 1

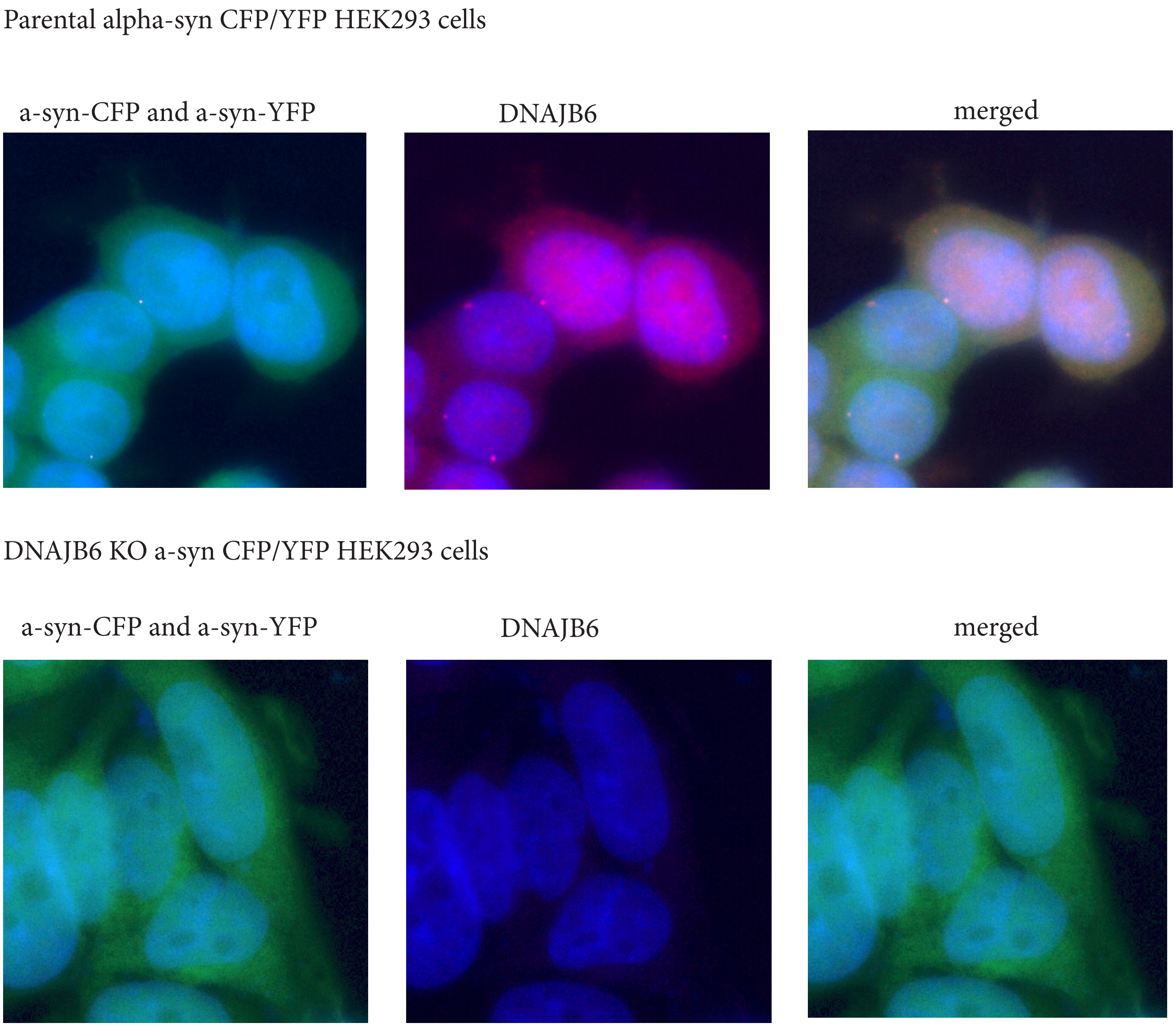

Supplementary figure 1: Representative images displaying staining of a-syn CFP/YFP HEK293 cells parental (A) or DNAJB6 KO (B), after staining with Rabbit anti-DNAJB6 antibody and Cy3 labeled goat anti rabbit secondary antibody.

Supplementary figure 2

Parental a-syn CFP/YFP HEK293

a-syn-CFP and a-syn-YFP DNAJB1 merged

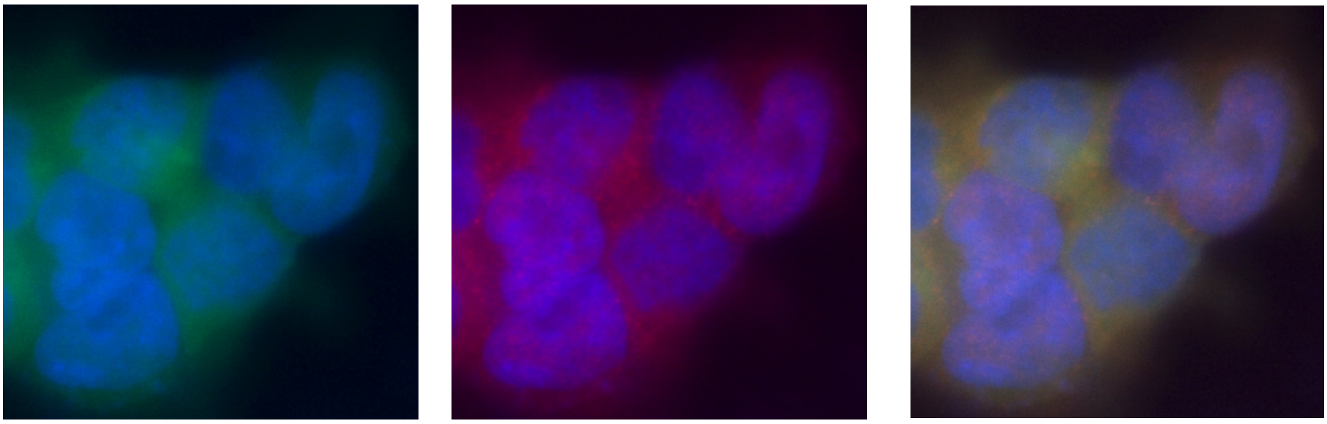

DNAJB1KO a-syn CFP/YFP HEK293

a-syn-CFP and a-syn-YFP DNAJB1 merged

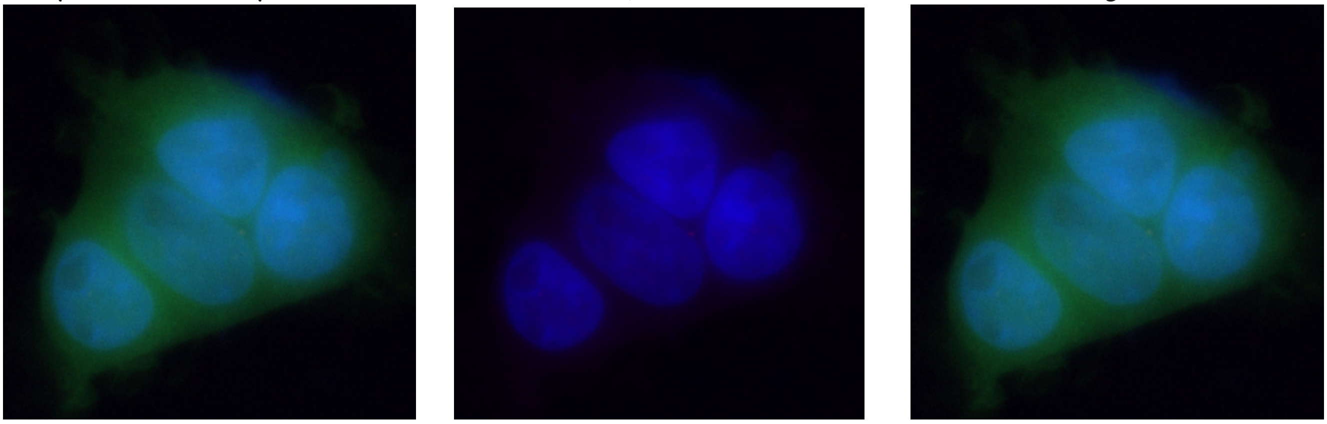

Supplementary figure 2: Representative images displaying staining of a-syn CFP/YFP HEK293 cells parental (A) or DNAJB1 KO (B), after staining with Rabbit anti-DNAJB6 antibody and Cy3 labeled goat anti rabbit secondary antibody.

Supplementary figure 3

A

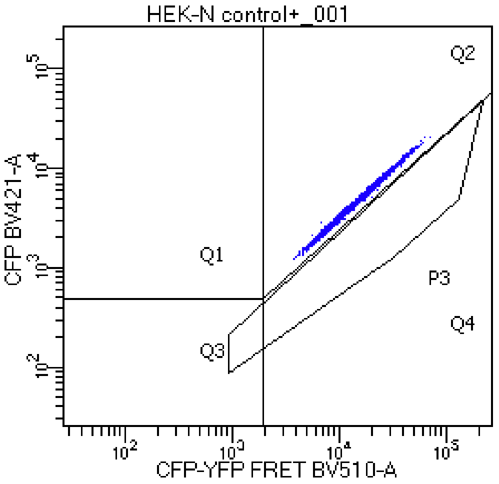

B

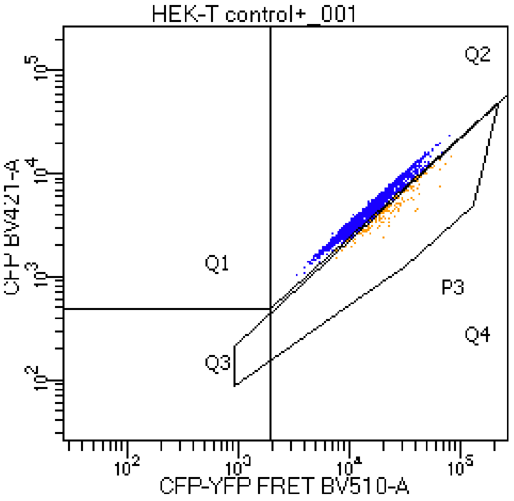

C

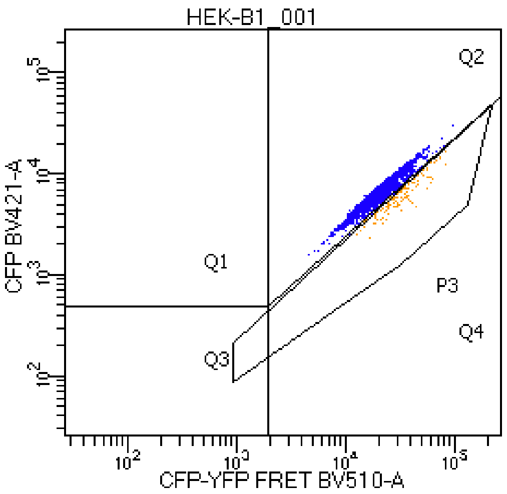

D

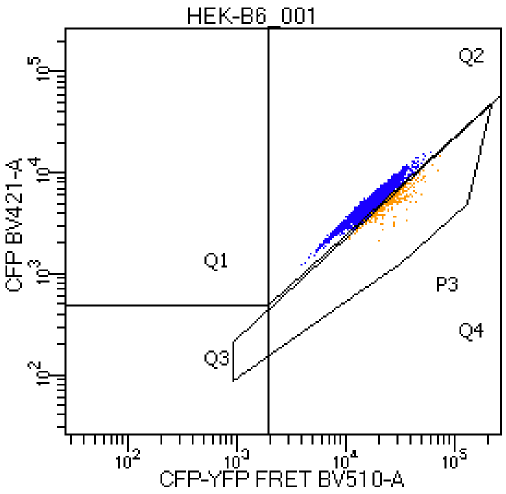

Supplementary figure 3: Representative images displaying the amount of FRET-positive cells. In (A) 0,0 % FRET positive cells was detected in a sample of HEK293 parental a-syn CFP/YFP cells in which aggregation was not induced by PFF's. In (B-D) a-syn aggregation was induced by a-syn PFF's: In (B) 1,7 % FRET positive cells were detected in parental cells, in (C) 1,3% of FRET positive DNAJB1 KO cells were detected and in (D) 2,7 % of FRET positive DNAJB6KO cells were detected . In all of these experiments a minimum of 10.000 cells were counted.

Supplementary figure 4

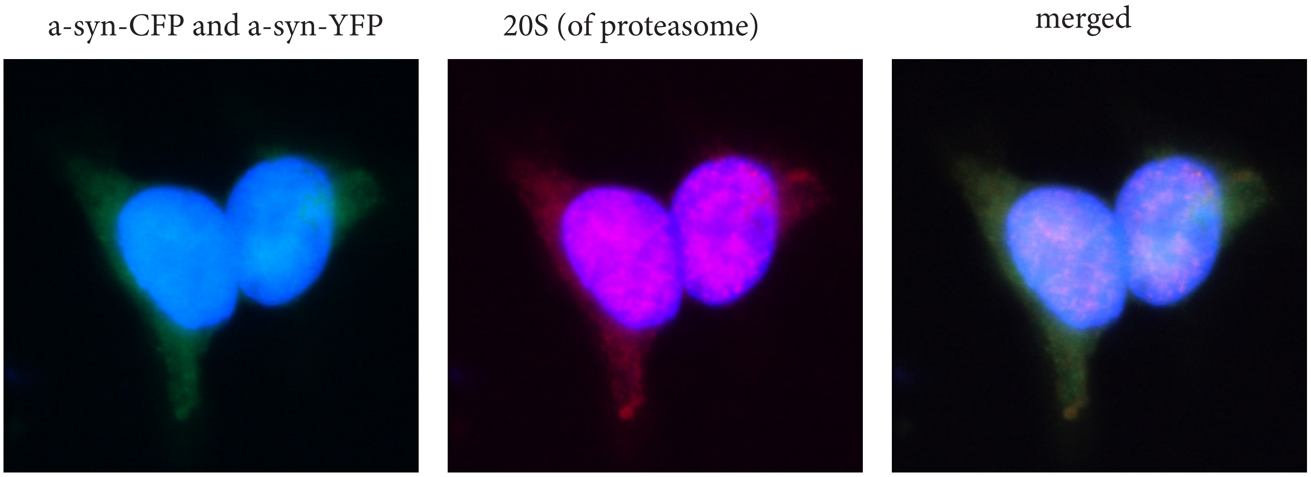

Supplementary figure 4: Representative images displaying staining of a-syn CFP/YFP HEK293 parental cells after staining with Rabbit anti-20S antibody and Cy3 labeled goat anti rabbit secondary antibody.
